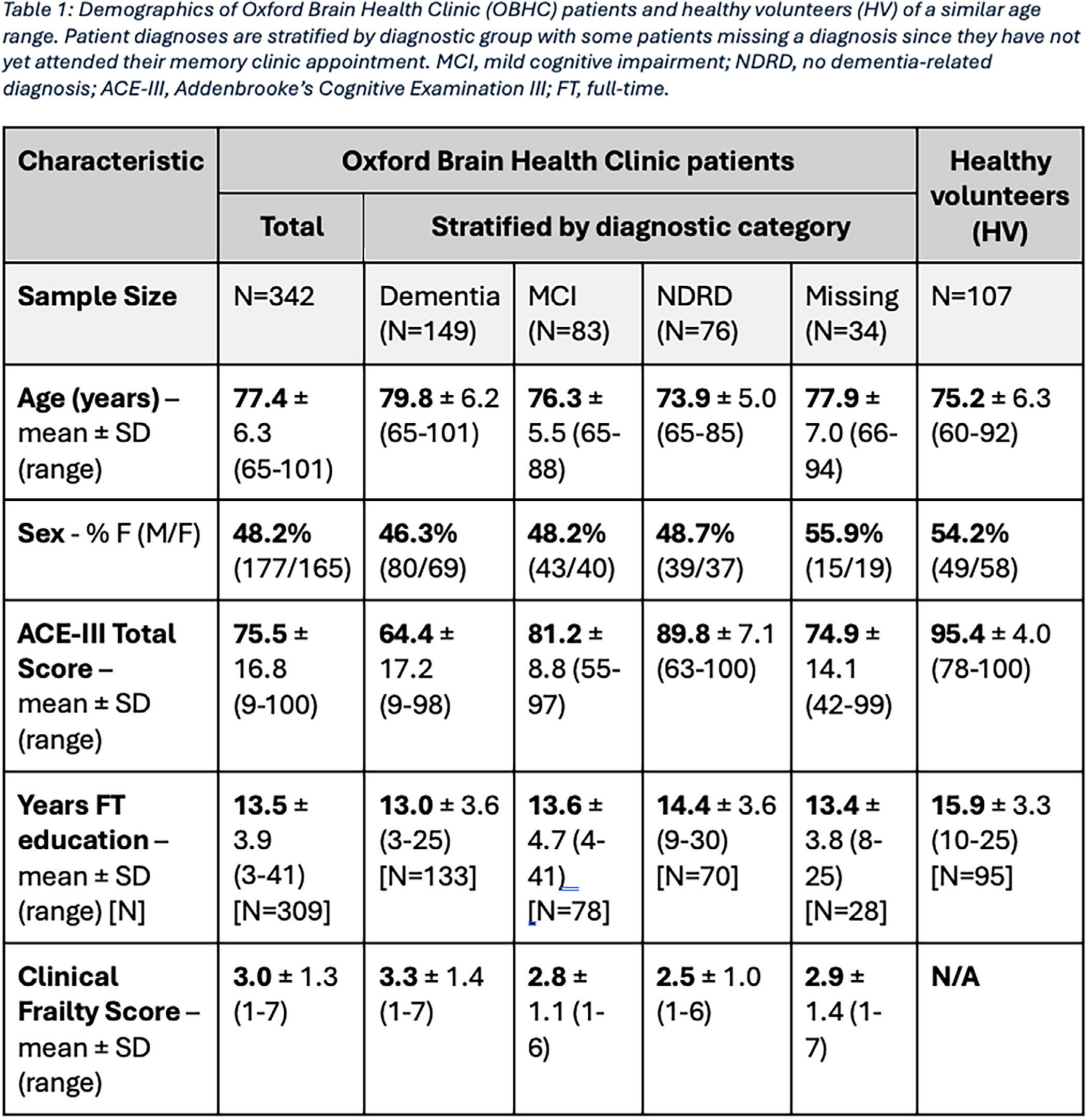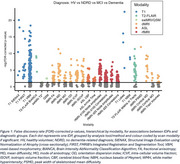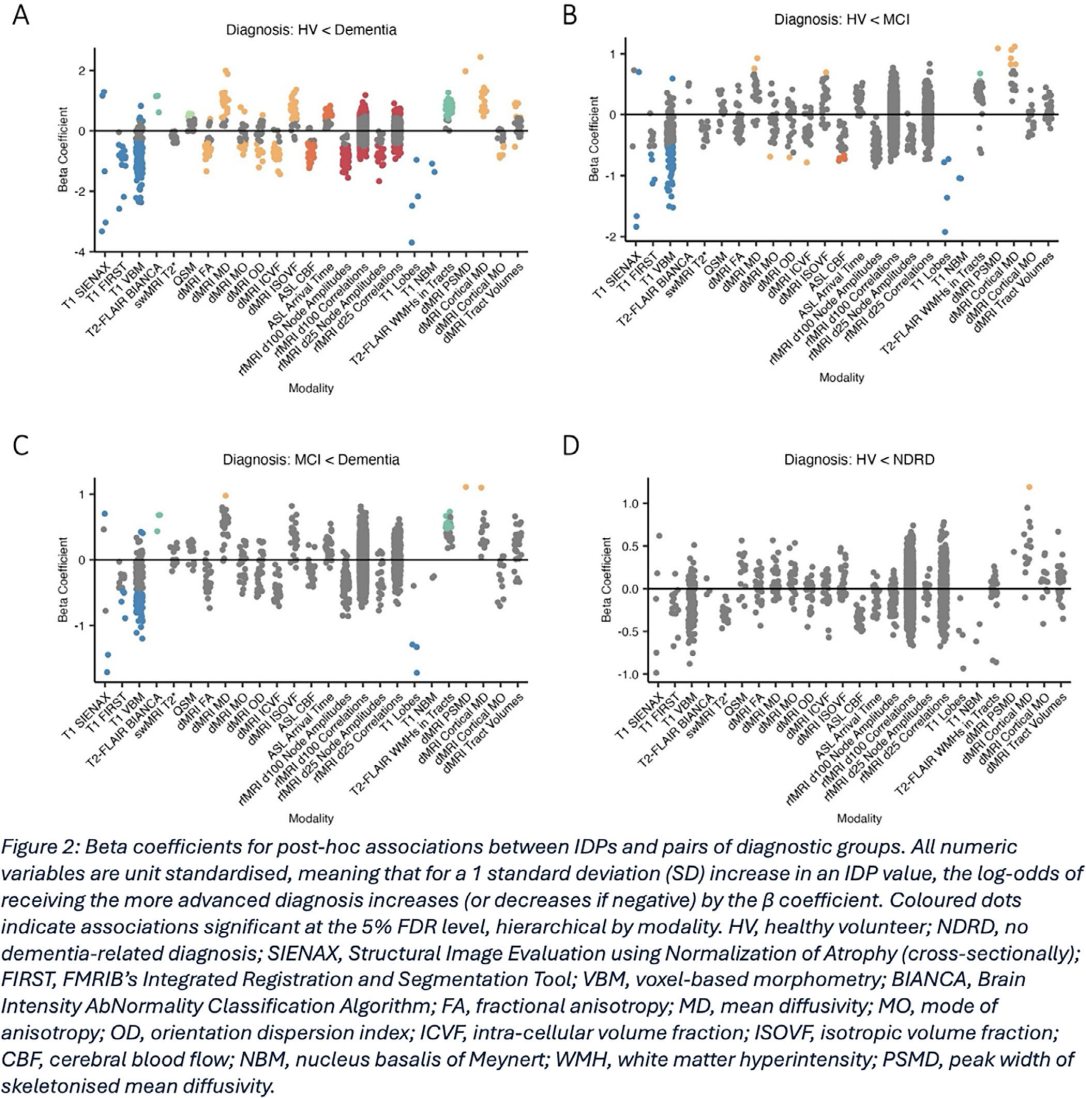# Multimodal MRI reveals distinct patterns of vascular and microstructural disruption across disease stages in the Oxford Brain Health Clinic

**DOI:** 10.1002/alz70862_110139

**Published:** 2025-12-23

**Authors:** Grace Gillis, Gaurav V Bhalerao, Jasmine Blane, Sameera Shabir, Luciana Maffei, Heidi Johansen‐Berg, Pieter M Pretorius, Lola Martos, Vanessa Raymont, Clare E Mackay, Ludovica Griffanti

**Affiliations:** ^1^ University of Oxford, Oxford, Oxfordshire UK; ^2^ Oxford Health NHS Foundation Trust, Oxford, Oxfordshire UK; ^3^ Oxford University Hospitals NHS Trust, Oxford UK

## Abstract

**Background:**

The Oxford Brain Health Clinic (OBHC) has assessed over 300 NHS memory clinic patients with a magnetic resonance imaging (MRI) protocol aligned with the UK Biobank. We also acquired the same data from over 100 healthy volunteers (HV) of a similar age range. This work explores multimodal patterns of imaging‐derived phenotypes (IDPs) across diagnostic groups in a real‐world memory clinic setting.

**Method:**

Scans from 342 OBHC patients and 107 HV (demographics in Table 1) were processed with an integrated quality control‐analysis pipeline optimised for memory clinic use (Gillis *et al.*, medRxiv, 2024). Subsequent diagnoses were extracted from electronic healthcare records and categorised as follows: dementia (ICD10 codes F00, F01, F02, F03), mild cognitive impairment (MCI ‐ F06.7), and no dementia‐related diagnoses (NDRD, including F10, F31, F32, F41). We performed ordinal regression analyses to test associations of IDPs with diagnoses, controlling for age, sex, head size, and applying hierarchical FDR correction.

**Result:**

IDPs from all 6 MRI modalities significantly differed across groups (Figure 1). Pairwise post‐hoc analyses revealed that healthy volunteers and dementia patients also significantly differed across all modalities (Figure 2A). In addition to structural changes, MCI patients had significantly higher cortical mean diffusivity, lower white matter integrity, and lower cerebral blood flow compared to HV (Figure 2B). Dementia patients had smaller volumes, localised increases in mean diffusivity, and more white matter hyperintensities (WMHs) than MCI patients (Figure 2C). Memory clinic patients who received no formal dementia‐related diagnosis did not have significantly different brain volumes compared to HV, but the left hippocampal mean diffusivity was significantly higher (Figure 2D).

**Conclusion:**

Thanks to the comprehensive multimodal MRI assessment offered in the OBHC, we observed distinct patterns of changes across the dementia spectrum. While structural IDPs may still provide best sensitivity, non‐conventional MRI may give further insights on mechanisms of neurodegeneration. Microstructural and perfusion changes may precede the formation of overt WMH lesions, supporting the possibility of diffusion MRI and perfusion imaging as early signatures alongside structural imaging. Increased mean diffusivity in the left hippocampus in NDRD might explain memory problems that led to the referral to memory clinic.